# Anatomical Variations and Morphometric Features of the Anterior Cerebral Artery: A Systematic Review and Meta-Analysis of 24,015 Cases

**DOI:** 10.3390/brainsci15121277

**Published:** 2025-11-28

**Authors:** Michał Bonczar, Kamil Możdżeń, Agnieszka Murawska, Julia Toppich, Patryk Ostrowski, Ahmed Elsaftawy, Anna Yevstifeieva, Mateusz Koziej, Magdalena Grzonkowska, Stanisław Orkisz, Andrzej Żytkowski, Piotr Wysocki, Michał Polguj, Grzegorz Wysiadecki

**Affiliations:** 1 Department of Anatomy, Jagiellonian University Medical College, 31-034 Kraków, Poland; michal.bonczar@student.uj.edu.pl (M.B.); kamil.mozdzen@student.uj.edu.pl (K.M.); agnieszka.murawska@student.uj.edu.pl (A.M.); julia.toppich@student.uj.edu.pl (J.T.); patryk.ostrowski@student.uj.edu.pl (P.O.); anna.yevstifeieva@uj.edu.pl (A.Y.); mateuszkoziej01@gmail.com (M.K.); 2 Youthoria, Youth Research Organization, 31-034 Kraków, Poland; 3 Department of Plastic and Hand Surgery, St. Jadwiga Śląska Hospital, 55-100 Trzebnica, Poland; ahmed.elsaftawy05@gmail.com; 4 Department of Normal Anatomy, The Ludwik Rydygier Collegium Medicum in Bydgoszcz, The Nicolaus Copernicus University in Toruń, 85-094 Bydgoszcz, Poland; m.grzonkowska@cm.umk.pl; 5 Department of Normal and Clinical Anatomy, Faculty of Medicine, University of Social Sciences in Lodz, 90-113 Łódź, Poland; orkisz.stanislaw@gmail.com (S.O.); andrzej.zytkowski.anat@gmail.com (A.Ż.); 6 Norbert Barlicki Memorial Teaching Hospital No. 1 of the Medical University of Lodz, 90-153 Łódź, Poland; 7 Faculty of Medicine, Medical University of Lodz, 90-419 Łódź, Poland; piotr.wysocki@stud.umed.lodz.pl; 8 Department of Normal and Clinical Anatomy, Chair of Anatomy and Histology, Medical University of Lodz, 90-752 Łódź, Poland; michal.polguj@umed.lodz.pl; 9 Laboratory of Neuroanatomy, Department of Normal and Clinical Anatomy, Chair of Anatomy and Histology, Medical University of Lodz, 90-752 Łódź, Poland

**Keywords:** anterior cerebral artery, brain, cerebral arteries, frontal lobe, neuroanatomy, neurosurgery

## Abstract

**Background/Objectives**: The anterior cerebral artery (ACA), as one of the terminal branches of the internal carotid artery, supplies the medial and superior portions of the frontal lobes as well as the anterior portions of the parietal lobes. The present meta-analysis aims to consolidate current knowledge regarding the anatomy and variations in the ACA, providing a comprehensive resource for physicians. **Methods:** To conduct this meta-analysis, we systematically searched prominent online medical databases, including PubMed, Scopus, Embase, Web of Science, Cochrane Library, and Google Scholar, to identify all studies that investigated the anatomy of the ACA. **Results:** The results of the present study were based on a total of 73 articles. In the aforementioned studies, a total of 24,015 patients were evaluated. The pooled mean total length of the A1 segment of the ACA, based on all evaluated cases, was 14.47 mm (SE = 0.28). The pooled mean total diameter of the A1 segment measured 2.00 mm on average (SE = 0.07). The overall pooled prevalence of the median ACA was 2.65% (95% CI: 1.57–3.99%). **Conclusions:** This systematic review and meta-analysis provide valuable insights into the anatomy and variations in the ACA. The current data may support clinicians and neurosurgeons in the management of cerebrovascular diseases and associated procedures, potentially enhancing procedural safety and therapeutic outcomes.

## 1. Introduction

**Figure 1:**
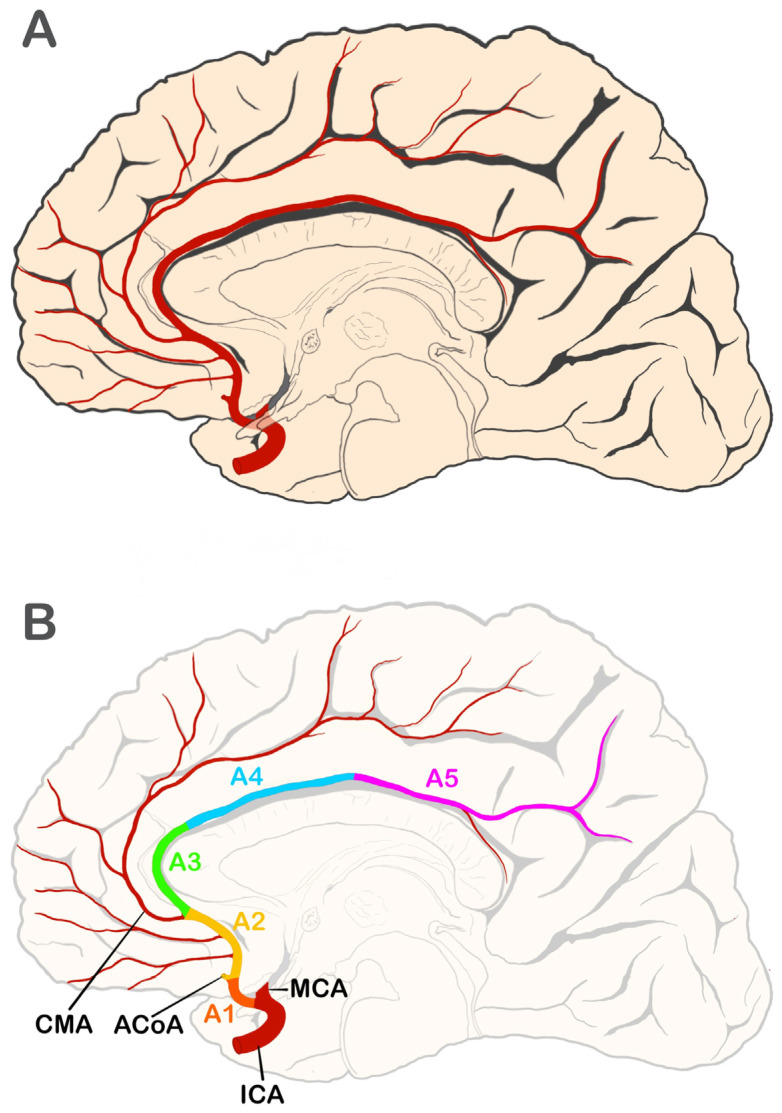
Anatomy and segments of the anterior cerebral artery (ACA). (**A**) Illustration showing general topographical relationships—medial view of the right hemisphere. (**B**) Schematic representation of ACA segments. ACoA—anterior communicating artery; CMA—callosomarginal artery; ICA—internal carotid artery; MCA—middle cerebral artery. ACA segments: A1—horizontal or precommunicating segment; A2—vertical, postcommunicating or infracallosal segment; A3—precallosal segment; A4—supracallosal segment; A5—postcallosal segment. Prepared by G. Wysiadecki.

The anterior cerebral artery (ACA) originates from the terminal bifurcation of the internal carotid artery and primarily supplies the medial and superior regions of the frontal lobes as well as the anterior part of the parietal lobe. It also provides arterial supply to parts of the corpus callosum and to deep cerebral structures, such as the basal ganglia [1]. From its origin, the ACA runs rostrally and medially toward the longitudinal cerebral fissure, where it connects with its counterpart through the anterior communicating artery. It continues to follow the path of the longitudinal cerebral fissure, curving around the genu of the corpus callosum, and then extends posteriorly along its body. As the ACA courses anterior to the genu of the corpus callosum, it typically bifurcates into its two terminal branches: the pericallosal and callosomarginal arteries. Near the splenium of the corpus callosum, these branches form anastomoses with the distal branches of the middle and posterior cerebral arteries [1,2,3]. Considering the distinct topography along its trajectory, the ACA is conventionally divided into five segments (A1–A5; see Figure 1). The precommunicating segment (A1) extends from the bifurcation of the internal carotid artery to the origin of the anterior communicating artery. This segment usually courses superior to the optic chiasm and optic nerves and inferior to the anterior perforated substance. The infracallosal segment (A2), also referred to as the vertical or postcommunicating segment, curves around the rostrum of the corpus callosum, running from the anterior communicating artery to the genu of the corpus callosum as far as the origin of the callosomarginal artery. The precallosal segment (A3) originates at the callosomarginal artery, wraps around the genu of the corpus callosum, and then turns posteriorly to ascend over the rostral portion of the corpus callosum body. The supracallosal segment (A4) travels along the dorsal surface of the corpus callosum anterior to the coronal suture, whereas the postcallosal segment (A5) extends posteriorly above the corpus callosum beyond the coronal suture [2]. Numerous variants of the ACA have been reported, particularly concerning its course and blood supply territory. Among the most frequently described are the median ACA (MedACA), the bihemispheric ACA (BihemACA), and the azygos ACA (AzACA) [4]. AzACA results from fusion of the right and left A2 segments into a single A2 trunk. Although uncommon, this configuration has been documented in several studies [5,6]. A BihemACA occurs when one of the A2 segments is hypoplastic or terminates early, causing the contralateral ACA to supply both hemispheres. Additionally, a MedACA refers to a third branch that supplies the medial surface of one or both hemispheres and can originate from the anterior communicating artery or the A1/A2 segments [4]. Adequate knowledge of the anatomy and variations in the ACA is important across clinical and neurosurgical practice, especially during the diagnosis of strokes, endovascular procedures, and interhemispheric surgeries [6,7,8,9]. Therefore, the present meta-analysis aims to consolidate current knowledge regarding the anatomy and variations in the ACA, providing a comprehensive resource for physicians. By synthesizing data from multiple studies, this study offers insights that may enhance surgical planning, improve clinical decision-making, and ultimately contribute to better outcomes in cerebrovascular interventions.

## 2. Materials and Methods

### 2.1. Search Strategy

To conduct this meta-analysis, we systematically searched prominent online medical databases, including PubMed, Scopus, Embase, Web of Science, Cochrane Library, and Google Scholar, to identify all studies that investigated the anatomy of the ACA. The comprehensive search was carried out in three distinct stages. In the first step, the following search terms were used in all databases: (anterior cerebral artery) AND ((anatomy) OR (topography) OR (morphology) OR (variation) OR (variant) OR (pattern) OR (branches) OR (origin) OR (diameter) OR (length)). No restrictions regarding date, language, article type, or text availability were applied. In the second step, the databases were searched again using another set of search phrases: (a) (anterior cerebral artery [Title/Abstract]) AND (anatomy [Title/Abstract]); (b) (anterior cerebral artery [Title/Abstract]) AND (topography [Title/Abstract]); (c) (anterior cerebral artery [Title/Abstract]) AND (morphology [Title/Abstract]); (d) (anterior cerebral artery [Title/Abstract]) AND (variations [Title/Abstract]). Each phrase was evaluated to determine its dependence on grammatical variations, and adjustments were made to tailor it to the specific requirements of each database. In the third stage, a manual search was conducted through the reference lists of the initially identified studies. The Preferred Reporting Items for Systematic Reviews and Meta-Analyses (PRISMA) guidelines were followed throughout the study. To ensure the highest quality of the findings, the Critical Appraisal Tool for Anatomical Meta-Analyses (CATAM) and the Anatomical Quality Assessment (AQUA) tools were used to evaluate the included studies [10,11,12].

### 2.2. Eligibility Assessment

**Figure 2:**
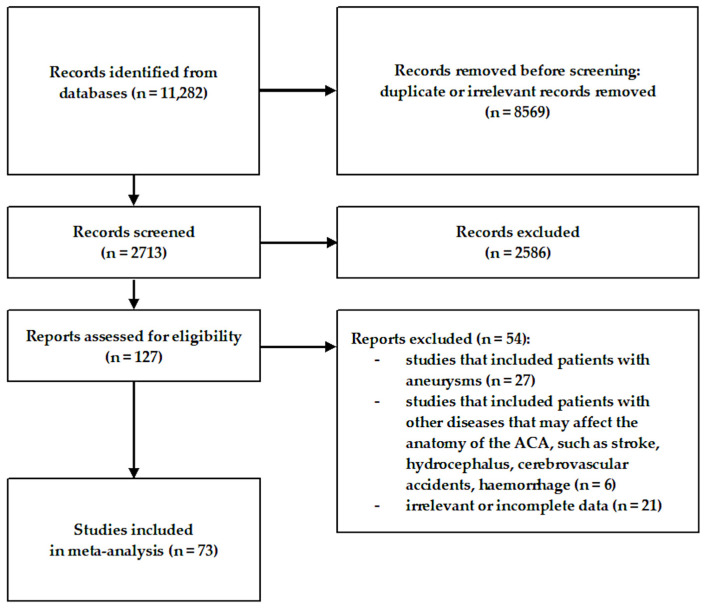
Flow diagram presenting the process of collecting data included in this meta-analysis.

A total of 11,282 articles were initially evaluated by two independent researchers. Of these, 8569 records were excluded for being irrelevant to the studied topic. Articles such as case reports, case series, conference reports, reviews, letters to the editor, and studies providing incomplete or irrelevant data were also excluded. The inclusion criteria encompassed original studies containing retrievable numerical data pertaining to the origin, diameter, length, or any other information relevant to the comprehensive anatomy, morphology, topography, or variations in the ACA. Ultimately, 73 studies were included in the present review [4,13,14,15,16,17,18,19,20,21,22,23,24,25,26,27,28,29,30,31,32,33,34,35,36,37,38,39,40,41,42,43,44,45,46,47,48,49,50,51,52,53,54,55,56,57,58,59,60,61,62,63,64,65,66,67,68,69,70,71,72,73,74,75,76,77,78,79,80,81,82,83,84]. The flow chart presenting the study inclusion process is shown in Figure 2.

### 2.3. Data Extraction

Data from eligible articles were extracted by two independent researchers. Qualitative data, such as methodology, year of publication, and country of origin, were assessed. In addition, quantitative data about the anatomy of the ACA were extracted. Any discrepancies between the reviewers were resolved through contact with the original study authors whenever possible or by consensus with a third reviewer.

### 2.4. Statistical Analysis

To carry out the meta-analyses, MetaXL (version 5.3; EpiGear International Pty Ltd., Wilston, QLD, Australia), alongside Comprehensive Meta-analysis software (version 4.0; Biostat, Inc., Englewood, NJ, USA), were applied. All statistical analyses were conducted using a random-effects model. Heterogeneity among the included studies was evaluated using the I^2^ statistic [85,86]. Statistical significance was assessed based on *p*-values and confidence intervals, with results considered significant when *p* < 0.05. When confidence intervals overlapped, the differences were treated as statistically nonsignificant. Interpretation of I^2^ values followed standard thresholds: 0–40% suggesting low or negligible heterogeneity, 30–60% indicating moderate heterogeneity, 50–90% reflecting substantial heterogeneity, and 75–100% representing considerable heterogeneity [85].

## 3. Results

### 3.1. General Characteristics

The results of the present study were based on a total of 73 articles. In these studies, a total of 24,015 patients were evaluated. The meta-analysis was performed only on data that met the required statistical criteria and were eligible to be analyzed without potential bias. Data from the remaining articles are described below. Most of the included articles were conducted in Asia (n = 24) and Europe (n = 20). The majority of the studies (n = 48) were based on cadaveric dissections, while the remaining (n = 26) were based on radiological images. Despite methodological differences, no statistically significant differences (*p* > 0.05) were found between the results of the radiological and cadaveric studies in the categories included in the meta-analysis. The characteristics of the included studies are presented in Table 1.

**Table 1:** Characteristics of included studies. CTA—Computed Tomography Angiography. MRI—Magnetic Resonance Imaging. MRA—Magnetic Resonance Angiography.

First Author	Year	Continent	Country	Method
Madkour, N.A.A. [82]	2023	Africa	Egypt	MRA
Luckrajh, J.S. et al. [81]	2022	Africa	South Africa	CTA
Riveros, A. et al. [80]	2022	South America	Chile	Cadavers
Giotta Lucifero, A. et al. [79]	2021	Europe	Italy	Cadavers
Beyhan, M. et al. [78]	2020	Asia	Turkey	CTA + MRA + MRI + DSA
Quijano Blanco, Y. & García Orjuela, D. [77]	2020	South America	Colombia	Cadavers
Sharma, S. et al. [76]	2020	Asia	India	CTA
Zaki, S.M. et al. [75]	2019	Africa	Egypt	MRA
Thenmozhi, A. et al. [74]	2019	Asia	India	Cadavers
Shatri, J. et al. [73]	2019	Europe	Germany	MRA
Canaz, H. et al. [72]	2018	Europe	Turkey	Cadavers
Jiménez-Sosa, M.S. et al. [71]	2017	South America	Mexico	CT
Ozturk, S. et al. [70]	2017	Europe	Turkey	MRI
Yu, L-H. et al. [69]	2017	Asia	China	Cadavers
Shatri, J. et al. [68]	2017	Europe	Germany	MRA
Cilliers, K. & Page, B.J. [67]	2017	Africa	South Africa	Cadavers
Cilliers, K. & Page, B.J. [4]	2016	Africa	South Africa	Cadavers
Karatas, A. et al. [66]	2016	Europe	Turkey	Cadavers
Shinde, S. & Shroff, G. [65]	2016	Asia	India	Cadavers
Aggarwal, N. et al. [64]	2016	Asia	India	MRA
Arat, Y. et al. [63]	2015	Asia	Turkey	CTA
d’Avella, E. et al. [62]	2015	Europe	Spain	Cadavers + CT + MR
Klimek-Piotrowska, W. et al. [61]	2015	Europe	Poland	Cadavers
Karatas, A. et al. [60]	2015	Europe	Turkey	CTA
Gunnal, S.A. et al. [59]	2013	Asia	India	Cadavers
Flores, B.C. et al. [58]	2013	North America	USA	CTA
Hamidi, C. et al. [57]	2013	Asia	Turkey	CTA
Kedia, S. et al. [56]	2013	Asia	India	Cadavers
Stefani, M.A. et al. [55]	2013	South America	Brasil	MRA
Aggarwal, N. et al. [54]	2012	Asia	India	MRA
Swetha, B. [53]	2012	Asia	India	Cadavers
Shi, W.-Y. et al. [52]	2014	Asia	China	MRI
Nordon, D. & Rodrigues Junior, O. [51]	2012	South America	Brasil	Cadavers
Maaly, M.A. & Ismail, A.A. [50]	2011	Africa	Egypt	MRA
Żurada, A. & Gielecki, J. [49]	2010	Europe	Poland	CT
Ozdogmus, O. et al. [48]	2008	Europe	Turkey	Cadavers
Kahilogullari, G. et al. [47]	2008	Asia	Turkey	Cadavers
Kapoor, K. et al. [46]	2008	Asia	India	Cadavers
Lehecka, M. et al. [45]	2008	Europe	Finland	CTA
Saidi, H. et al. [44]	2008	Africa	Kenya	Cadavers
Mandiola, E. et al. [43]	2007	South America	Chile	Cadavers
Tao, X. & Yu, X.J. [42]	2006	Asia	China	Cadavers
Forero, P. [83]	2006	South America	Colombia	Cadavers
Ugur, H.C. et al. [41]	2006	Asia	Turkey	Cadavers
Pai, S.B. et al. [40]	2005	Asia	India	Cadavers
Ugur, H.C. et al. [39]	2005	Asia	Turkey	Cadavers
Paul, S. & Mishra, S. [84]	2004	Asia	India	Cadavers
Kulenović, A. et al. [38]	2003	Europe	Croatia	Cadavers
Avci, E. et al. [37]	2001	North America	USA	Cadavers
Stefani, M.A. et al. [36]	2000	South America	Brazil	Cadavers
Serizawa, T. et al. [35]	1997	Asia	Japan	Cadavers
Macchi, C. et al. [34]	1996	Europe	Italy	MRA
Piepgras, A. et al. [33]	1993	Europe	Germany	Cadavers
Sanders, W.P. et al. [32]	1993	North America	USA	Agniograms
van der Zwan, A. et al. [31]	1992	North America	USA	Cadavers
Krabbe-Hartkamp, M.J. et al. [30]	1988	Europe	The Netherlands	MRA
Gomes, F.B. et al. [29]	1986	North America	USA	Cadavers
Orlandini, G.E. & Ruggiero, C. [28]	1985	Europe	Italy	Cadavers
Kamath, S. [27]	1980	Asia	India	Cadavers
Huber, P. & Braun, J. [26]	1980	Europe	Switzerland	Angiograms
Perlmutter, D. & Rhoton, A.L. [25]	1978	North America	USA	Cadavers
Tulleken, C.A.F. [24]	1978	Europe	The Netherlands	Cadavers
Ozaki, T. et al. [23]	1977	Asia	Japan	Cadavers
Perlmutter, D. & Rhoton, A.L. [22]	1976	North America	USA	Cadavers
Dunker, R.O. & Harris, A.B. [21]	1976	North America	USA	Cadavers
Ring, B.A. & Waddington, M.M. [20]	1968	North America	USA	Cadavers
Wollschlaeger, G. et al. [19]	1967	South America	Colombia	Angiograms
Lemay, M. & Gooding, C.A. [18]	1966	North America	USA	Arteriograms
Fisher, C. [17]	1965	North America	USA	Cadavers
Jain, K.K. [16]	1964	Asia	India	Cadavers
Murray, K.D. [15]	1964	Australia	South Australia	Cadavers
Baptista, A.G. [14]	1963	South America	Brazil	Cadavers
Windle, B.C. [13]	1888	Europe	England	Cadavers

### 3.2. Length of the A1 Segment of the ACA

The pooled mean total length of the A1 segment of the ACA, based on all evaluated cases, was 14.47 mm (SE = 0.28). Based solely on cadaveric studies, the pooled mean length was 14.61 mm (SE = 0.64), while for radiological images, it was 14.11 mm (SE = 0.20). The complete results of this part of the meta-analysis, along with remaining data from the literature not included in the analysis, are presented in Table 2.

**Table 2:** Statistical results of this meta-analysis regarding the length of the A1 segment of the Anterior Cerebral Artery (ACA). (*)—The results of this part of the analysis were based on the following studies: Kamath, S. [27]; Gomes, F.B. et al. [29]; Tao, X. & Yu, X.J. [42]; Mandiola, E. et al. [43]; Aggarwal, N. et al. [54]; d’Avella, E. et al. [62]; Karatas, A. et al. [60]; Karatas, A. [et al. [66]; Yu, L.-H. et al. [69]; Canaz, H. et al. [72]; Shatri, J. et al. [73]; Sharma, S. et al. [76]; Luckrajh, J.S. et al. [81]; Riveros, A. et al. [80]. (**)—The rest of the results were gathered from the literature, but not included in the analysis due to insufficient data or bias prevention.

Category	Pooled Mean	Standard Error	Variance	Lower Limit	Upper Limit	Z-Value	*p*-Value
A1 Segment Length [mm] *							
Total Length [Overall]	14.47	0.28	0.08	13.92	15.03	51.44	0.00
Total Length [Cadavers]	14.61	0.64	0.41	13.36	15.87	22.84	0.00
Total Length [Radiological Studies]	14.11	0.20	0.04	13.71	14.50	70.33	0.00
Right ACA Length [Overall]	14.49	0.26	0.07	13.98	15.00	55.72	0.00
Right ACA Length [Cadavers]	14.46	0.40	0.16	13.68	15.25	36.04	0.00
Right ACA Length [Radiological Studies]	14.68	0.46	0.21	13.77	15.58	31.71	0.00
Left ACA Length [Overall]	14.19	0.27	0.07	13.66	14.71	52.80	0.00
Left ACA Length [Cadavers]	14.08	0.34	0.11	13.42	14.74	41.92	0.00
Left ACA Length [Radiological Studies]	14.61	0.59	0.35	13.45	15.77	24.66	0.00
Other Results from the Literature **							
Author				Data			
Total Length [mm]							
Giotta Lucifero, A. et al. [79]				Mean = 12.7			
Shatri, J. et al. [68]				Mean = 13.96; Minimum = 1.4			
Avci, E. et al. [37]				Minimum = 7.07; Maximum = 15.5			
Perlmutter, D. & Rhoton, A.L. [25]				Mean = 13; Minimum = 7; Maximum = 18			
Perlmutter, D. & Rhoton, A.L. [22]				Mean = 12.7; Minimum = 7.2; Maximum = 18			
Dunker, R.O. & Harris, A.B. [21]				Median = 13			
Right ACA Length [mm]							
Thenmozhi, A., 2019 [74]				Mean = 14.44			
Shinde, S. & Shroff, G. [65]				Mean = 12			
Kedia, S. et al. [56]				Mean = 12.09			
Pai, S.B. et al. [40]				Mean = 14.6			
Orlandini, G.E. & Ruggiero, C. [28]				Mean = 14.1			
Murray, K.D. [15]				Mean = 13.2			
Left ACA Length [mm]							
Thenmozhi, A., 2019 [74]				Mean = 13.6			
Shinde, S. & Shroff, G. [65]				Mean = 13			
Kedia, S. et al. [56]				Mean = 12			
Pai, S.B. et al. [40]				Mean = 14.5			
Orlandini, G.E. & Ruggiero, C. [28]				Mean = 13.6			
Murray, K.D. [15]				Mean = 12.9			

### 3.3. Diameter of the A1 Segment of the ACA

The pooled mean diameter of the A1 segment of the ACA, based on all evaluated cases, was 2.00 mm (SE = 0.07). In cadaveric studies, the pooled mean diameter was 1.86 mm (SE = 0.15), while in radiological studies, it was 2.12 mm (SE = 0.09). The complete results of this part of the meta-analysis, together with additional data from the literature, are presented in Table 3.

**Table 3:** Statistical results of this meta-analysis regarding the diameter of the A1 segment of the Anterior Cerebral Artery (ACA). (*)—The results of this part of the analysis were based on the following studies: Kamath, S. [27]; Gomes, F.B. et al. [29]; Piepgras, A. et al. [33]; Stefani, M.A. et al. [36]; Tao, X. & Yu, X.J. [42]; Mandiola, E. et al. [43]; Ozdogmus, O. et al. [48]; Maaly, M.A. & Ismail, A.A. [50]; Shi, W.-Y. et al. [52]; Flores, B.C. et al. [58]; Stefani, M.A. et al. [55]; Arat, Y. et al. [63]; Klimek-Piotrowska, W. et al. [61]; Karatas, A. et al. [60]; Karatas, A. et al. [66]; Aggarwal, N. et al. [64]; Jiménez-Sosa, M.S. et al. [71]; Shatri, J. et al. [68]; Canaz, H. et al. [72]; Shatri, J. et al. [73]; Sharma, S. et al. [76]; Zaki, S.M. et al. [75]; Riveros, A. et al. [80]; Luckrajh, J.S. et al. [81]. (**)—The rest of the results were gathered from the literature, but not included in the analysis due to insufficient data or bias prevention.

Category	Pooled Mean	Standard Error	Variance	Lower Limit	Upper Limit	Z-Value	*p*-Value
A1 Segment Diameter [mm] *							
Total Diameter [Overall]	2.00	0.07	0.01	1.86	2.14	27.58	0.00
Total Diameter [Cadavers]	1.86	0.15	0.02	1.57	2.15	12.55	0.00
Total Diameter [Radiological Studies]	2.12	0.09	0.01	1.95	2.29	24.28	0.00
Right ACA Diameter [Overall]	2.04	0.05	0.00	1.94	2.13	44.17	0.00
Right ACA Diameter [Cadavers]	2.00	0.10	0.01	1.80	2.21	19.43	0.00
Right ACA Diameter [Radiological Studies]	2.09	0.05	0.00	1.99	2.19	40.30	0.00
Left ACA Diameter [Overall]	2.17	0.06	0.00	2.06	2.28	38.66	0.00
Left ACA Diameter [Cadavers]	2.11	0.11	0.01	1.88	2.33	18.36	0.00
Left ACA Diameter [Radiological Studies]	2.25	0.07	0.01	2.11	2.39	31.61	0.00
Other Results from the Literature **							
Author			Data				
Total Diameter [mm]							
Giotta Lucifero, A. et al. [79]			Mean = 2.5				
Krabbe-Hartkamp, M.J. et al. [30]			Mean = 2.2				
Perlmutter, D. & Rhoton, A.L. [22]			Mean = 2.6				
Right ACA Diameter [mm]							
Quijano Blanco, Y. & García Orjuela, D. [77]			Mean = 2.29				
Shinde, S. & Shroff, G. [65]			Mean = 2.1				
Kedia, S. et al. [56]			Mean = 2.32				
Forero, P, 2006 [83]			Mean = 2.21				
Pai, S.B. et al. [40]			Mean = 2.8				
Avci, E. et al. [37]			Mean = 1.9				
Krabbe-Hartkamp, M.J. et al. [30]			Mean = 2.3				
Left ACA Diameter [mm]							
Quijano Blanco, Y. & García Orjuela, D. [77]			Mean = 2.33				
Shinde, S. & Shroff, G. [65]			Mean = 2.4				
Kedia, S. et al. [56]			Mean = 2.36				
Forero, P, 2006 [83]			Mean = 1.16				
Pai, S.B. et al. [40]			Mean = 2.9				
Avci, E. et al. [37]			Mean = 2.1				
Krabbe-Hartkamp, M.J. et al. [30]			Mean = 2.2				

### 3.4. Length of the A2 Segment of the ACA

The pooled mean length of the right A2 segment of the ACA, based solely on radiological studies, was 18.39 mm (SE = 6.31), while the corresponding value for the left ACA was 18.34 mm (SE = 6.72). Reported values in the literature for the total length of the A2 segment range from 4.42 mm to 69.63 mm. Full results of this part of the meta-analysis, along with additional literature data, are presented in Table 4.

**Table 4:** Statistical results of this meta-analysis regarding the length of the A2 segment of the Anterior Cerebral Artery (ACA). (*)—The results of this part of the analysis were based on the following studies: Luckrajh, J.S. et al. [81]; Żurada, A. & Gielecki, J. [49] (**)—The rest of the results were gathered from the literature, but not included in the analysis due to insufficient data or bias prevention.

Category	Pooled Mean	Standard Error	Variance	Lower Limit	Upper Limit	Z-Value	*p*-Value
A2 Segment Length [mm] *							
Right ACA Length [Radiological Studies]	18.39	6.31	39.88	6.02	30.77	2.91	0.00
Left ACA Length [Radiological Studies]	18.34	6.72	45.16	5.17	31.51	2.73	0.01
Other Results from the Literature **							
Author				Data			
Total Length [mm]							
Quijano Blanco, Y. & García Orjuela, D. [77]				Mean = 69.63			
Yu, L.-H. et al. [69]				Mean = 4.42; Standard Deviation = 1.78			
d’Avella, E. et al. [62]				Mean = 7; Standard Deviation = 1			
Żurada, A. & Gielecki, J. [49]				Mean = 11.83; Standard Deviation = 4.89			
Perlmutter, D. & Rhoton, A.L. [25]				Mean = 28			
Right ACA Length [mm]							
Canaz, H. et al. [72]				Mean = 18.83; Standard Deviation = 3.18			
Cilliers, K. & Page, B.J. [4]				Mean = 19.1			
Swetha, B. [53]				Mean = 38.89			
Left ACA Length [mm]							
Canaz, H. et al. [72]				Mean = 18.73; Standard Deviation = 3.02			
Cilliers, K. & Page, B.J. [4]				Mean = 19.5			
Swetha, B. [53]				Mean = 41.01			

### 3.5. Diameter of the A2 Segment of the ACA

The pooled mean diameter of the A2 segment of the ACA, based solely on radiological studies, was 1.76 mm (SE = 0.07). The pooled mean diameters of the right and left A2 segments, based on all evaluated cases, were 1.87 mm (SE = 0.08) and 1.87 mm (SE = 0.07), respectively. Based on cadaveric studies alone, the pooled mean diameter was 2.16 mm (SE = 0.32) for the right ACA and 2.11 mm (SE = 0.28) for the left ACA. The full results of this part of the meta-analysis, along with other literature data, are presented in Table 5.

**Table 5:** Statistical results of this meta-analysis regarding the diameter of the A2 segment of the Anterior Cerebral Artery (ACA). (*)—The results of this part of the analysis were based on the following studies: Gomes, F.B. et al. [29]; Żurada, A. & Gielecki, J. [49]; Flores, B.C. et al. [58]; Jiménez-Sosa, M.S. et al. [71]; Canaz, H. et al. [72]; Luckrajh, J.S. et al. [81]. (**)—The rest of the results were gathered from the literature, but not included in the analysis due to insufficient data or bias prevention.

Category	Pooled Mean	Standard Error	Variance	Lower Limit	Upper Limit	Z-Value	*p*-Value
A2 Segment Diameter [mm] *							
Total Diameter [Radiological Studies]	1.76	0.07	0.00	1.63	1.89	26.74	0.00
Right ACA Diameter [Overall]	1.87	0.08	0.01	1.70	2.03	22.59	0.00
Right ACA Diameter [Cadavers]	2.16	0.32	0.10	1.53	2.79	6.76	0.00
Right ACA Diameter [Radiological Studies]	1.71	0.12	0.01	1.48	1.95	14.27	0.00
Left ACA Diameter [Overall]	1.87	0.07	0.00	1.74	2.00	28.04	0.00
Left ACA Diameter [Cadavers]	2.11	0.28	0.08	1.56	2.65	7.53	0.00
Left ACA Diameter [Radiological Studies]	1.74	0.14	0.02	1.47	2.02	12.45	0.00
Other Results from the Literature **							
Author				Data			
Total Diameter [mm]							
Cilliers, K. & Page, B.J. [4]				Mean = 2.3			
Ugur, H.C. et al. [41]				Mean = 2.75			
Stefani, M.A. et al. [36]				Mean = 2.6; Standard Deviation = 0.3			
Right ACA Diameter [mm]							
Quijano Blanco, Y. & García Orjuela, D. [77]				Mean = 1.98			
Cilliers, K. & Page, B.J. [4]				Mean = 2.3			
Swetha, B. [53]				Mean = 2			
Forero, P. [83]				Mean = 2.54			
Left ACA Diameter [mm]							
Quijano Blanco, Y. & García Orjuela, D. [77]				Mean = 2.21			
Cilliers, K. & Page, B.J. [4]				Mean = 2.4			
Swetha, B. [53]				Mean = 2.01			
Forero, P. [83]				Mean = 2.43			

### 3.6. Anomalous ACA

The overall pooled prevalence of the azygos ACA was 1.32% (95% CI: 0.84–1.89%). The pooled prevalence of the median ACA was 2.65% (95% CI: 1.57–3.99%), and of the bihemispheric ACA was 1.12% (95% CI: 0.57–1.83%). Interestingly, in all categories, the prevalence rates based on cadaveric studies were higher than those based on radiological studies. Although no statistically significant differences were found (*p* > 0.05) due to the wide confidence intervals, these results may suggest that certain anatomical variations in the ACA may remain undetected through radiological methods. Full results are shown in Table 6.

**Table 6:** Statistical results of this meta-analysis regarding the pooled prevalence of anomalous Anterior Cerebral Artery (ACA). The presented results are based on the following studies: Windle, B.C. [13]; Baptista, A.G. [14]; Jain, K.K. [16]; Fisher, C. [17]; Lemay, M. & Gooding, C.A. [18]; Wollschlaeger, G. et al. [19]; Ring, B.A. & Waddington, M.M. [20]; Dunker, R.O. & Harris, A.B. [21]; Ozaki, T. et al. [23]; Tulleken, C.A.F. [24]; Huber, P. & Braun, J. [26]; van der Zwan, A. et al. [31]; Sanders, W.P. et al. [32]; Macchi, C. et al. [34]; Serizawa, T. et al. [35]; Stefani, M.A. et al. [36]; Avci, E. et al. [37]; Kulenović, A. et al. [38]; Paul, S. & Mishra, S. [84]; Ugur, H.C. et al. [39]; Ugur, H.C. et al. [41]; Kahilogullari, G. 2008; Kapoor, K. et al. [46]; Saidi, H. et al. [44]; Lehecka, M. et al. [45]; Nordon, D. & Rodrigues Junior, O. [51]; Shi, W.-Y. et al. [52]; Gunnal, S.A. et al. [59]; Cilliers, K. & Page, B. [87]; Kedia, S. et al. [56]; Hamidi, C. et al. [57]; Stefani, M.A. et al. [55]; Cilliers, K. & Page, B.J. [4]; Jiménez-Sosa, M.S. et al. [71]; Shatri, J. et al. [73]; Madkour, N.A.A. [82]. LCI—lower confidence interval. HCI—higher confidence interval. Q—Cochran’s Q.

Category	Total n	Pooled Prevalence	LCI	HCI	Q	I^2^
Azygos ACA						
Overall	21,795	1.32%	0.84%	1.89%	199.52	82.46
Cadavers	2563	1.67%	0.81%	2.82%	54.80	61.68
Radiological Studies	19,232	0.97%	0.50%	1.60%	111.37	88.33
Median ACA						
Overall	21,795	2.65%	1.57%	3.99%	694.12	94.96
Cadavers	2563	4.56%	2.35%	7.41%	146.14	85.63
Radiological Studies	19,232	0.82%	0.28%	1.63%	210.74	93.83
Bihemispheric ACA						
Overall	21,795	1.12%	0.57%	1.83%	384.19	90.89
Cadavers	2563	1.95%	0.45%	4.32%	188.15	88.84
Radiological Studies	19,232	0.33%	0.07%	0.74%	114.92	88.69

### 3.7. Other Results from the Literature

The total length of the ACA, without segmentation, was measured by Ozturk et al., 2017 [70], with values ranging from 32 mm to 42 mm and a mean of 37.5 mm.

The comparison of A1 segment thickness between the right and left sides was studied by Ozaki et al., 1977 [23]. In 118 cases, the right and left ACAs were equally thick; in 8 cases, the right ACA was thicker, and in another 8, the left ACA was thicker.

The total length of the A3 segment was reported in two articles [25,67], with mean values ranging from 36.3 mm to 41.0 mm. Differences between the right and left ACA were addressed in two studies [53,67]; the mean right A3 length ranged from 37.2 mm to 40.39 mm, while the left ranged from 35.7 mm to 39.37 mm.

The diameter of the A3 segment was established in one study [67] and reported to be 2.1 mm. However, two articles [53,67] examined differences between the right and left sides, with reported values ranging from 1.09 mm to 2.00 mm for the right A3 and from 1.07 mm to 2.2 mm for the left A3.

The total length of the A4 segment was measured in two studies [25,67], with mean values ranging from 25.4 mm to 27.0 mm. A difference between the right and left ACA was reported in one article [67], where the mean right A4 length was 26.1 mm and the left 24.5 mm.

The diameter of the A4 segment was studied in only one article [67], which found an overall value of 1.6 mm, with right and left sides measured at 1.6 mm and 1.7 mm, respectively.

Azygos ACA is a vascular anomaly characterized by the formation of a single A2 segment in the midline, resulting from the persistence of the embryonic median artery of the corpus callosum [78]. The occurrence of azygos ACA types was examined in two studies [59,78], each using different classification methods. According to Beyhan et al., Type A (branching at the root of the A2 segment) occurred in 1.75% of patients, Type B (branching at the genu of the corpus callosum) in 7.02%, Type C (branching between the genu and mid-body) in 84.21%, and Type D (branching after the mid-body) in 7.02%. According to Gunnal et al., Type 1 (classical azygos ACA) occurred in 2.70% of patients, Type 2 (short median stem) in 1.80%, Type 3 (two A2 segments, one of which is short and terminates early) in 3.60%, Type 4 (azygos pericallosal artery) in 2.70%, and Type 5 (third azygos median A2 artery) in 0.90%. The first percentages refer to the number of azygos ACA cases studied, while the second refer to the total number of patients.

The mean diameter of the median and bihemispheric ACA was studied by Cilliers et al., 2016 [4], with both variations having a mean diameter of 1.8 mm and standard deviations of 0.3 and 0.4, respectively.

Atypical origins of the ACA were analyzed in one study [4], which reported that the ACA originated from the superior internal parietal artery in 7.30% of cases, from the paracentral lobule artery in 3.70%, from the inferior internal parietal artery in 3.30%, from the middle internal frontal artery in 0.70%, and from the posterior internal frontal artery in 0.70%.

## 4. Discussion

This meta-analysis provides a comprehensive overview of the anatomy and variations in the ACA, consolidating data from 24,015 cases. The findings enhance our understanding of ACA morphology, particularly its critical role in cerebrovascular and neurosurgical procedures.

The pooled mean length of the A1 segment was 14.47 mm across all studies, with slight differences between cadaveric (14.61 mm) and radiological (14.11 mm) data. Although minor, this variation may reflect methodological differences in measurement precision between dissection and imaging studies. These results are relatively consistent with previously published literature, such as the studies by Luckrajh et al. [81] and Sharma et al. [76]. The A1 segment’s diameter also showed variability, with a pooled mean of 2.00 mm overall, slightly smaller in cadaveric studies (1.86 mm) and larger in radiological studies (2.12 mm).

The A2 segment demonstrated more variability, with pooled mean lengths of 18.39 mm (right ACA) and 18.34 mm (left ACA) in radiological studies. The substantial standard errors (6.31 mm for the right and 6.72 mm for the left) indicate high individual variability, as confirmed by studies such as those by Quijano Blanco et al. [77] and Yu et al. [69], which reported a wide range from 4.42 mm to over 69 mm. Moreover, the pooled mean diameter of the A2 segment was 1.76 mm. This wide range highlights the morphometric variability of the A2 segment. These findings are significant because precise knowledge of ACA measurements is critical when treating aneurysms with either surgical clipping or endovascular techniques, where even slight anatomical differences may affect outcomes [88,89].

As previously discussed, anomalies of the ACA are classified into three types: AzACA, MedACA, and BihemACA. The present meta-analysis is the first to report pooled prevalence rates of these anomalies, which are essential not only anatomically but also in clinical practice [4]. The pooled prevalence of the AzACA was 1.32%, with a higher rate in cadaveric studies (1.67%) compared to radiological studies (0.97%). This variation can complicate neurosurgical procedures, as the AzACA often supplies both cerebral hemispheres, meaning damage or occlusion may result in bilateral ischemia [78]. Identifying this variation preoperatively is therefore essential to prevent potentially catastrophic outcomes.

The pooled prevalence of the MedACA was 2.65%, with a significantly higher prevalence in cadaveric studies (4.56%) than in radiological studies (0.82%). Detection of this anomaly is particularly important in cases involving interhemispheric tumors or vascular malformations approached via the interhemispheric route [90]. The discrepancy between cadaveric and radiological data suggests that some anomalies may be missed during clinical imaging, highlighting the need for more detailed radiological evaluations.

The BihemACA had the lowest pooled prevalence at 1.12% overall, again higher in cadaveric studies (1.95%) compared to radiological studies (0.33%). Similarly to the AzACA, the BihemACA—where one ACA supplies both hemispheres—poses a significant risk during surgery or endovascular intervention [91,92]. Inadvertent damage or occlusion may result in bilateral cerebral infarction, making its identification crucial in surgical planning [4].

The anatomical variations and morphometric properties of the ACA demonstrated in this study have several clinical implications. The variability in ACA length and diameter necessitates precision in diagnostic imaging and surgical intervention. For instance, aneurysms frequently occur at ACA bifurcation points, and the presence of an AzACA or BihemACA may alter the approach to endovascular coiling or surgical clipping. In the case of an AzACA, the fusion of the two A2 segments into a single vessel means that both hemispheres may rely on one artery. This elevates the risk during aneurysm treatment, as any compromise to the AzACA could lead to bilateral ischemia [6,87]. The surgeon must consider altered hemodynamics and ensure sufficient collateral circulation during and after the procedure.

Similarly, identifying a MedACA can influence decisions during interhemispheric surgery, as this vessel may supply vital areas of the medial frontal cortex. The MedACA may follow an unusual course, making it important for surgeons to carefully navigate around it to avoid damage. In procedures such as corpus callosotomy or tumor resection, an unrecognized MedACA may lead to ischemic complications if injured [8,93,94]. Detailed preoperative imaging is therefore essential to map its anatomy and ensure safe outcomes.

Understanding ACA variability also has implications for ischemic stroke management. Recognizing ACA anomalies may help predict the extent of cerebral ischemia and guide treatment strategies [95].

This study acknowledges certain limitations, including potential biases due to the heterogeneous nature of the source data. The reliability of findings is constrained by differences across studies, and some analyses could not be performed due to insufficient consistent data. Nonetheless, this meta-analysis offers a robust estimation of ACA anatomy based on literature that meets the standards of evidence-based anatomical research.

## 5. Conclusions

This systematic review and meta-analysis provides valuable insights into the anatomy and variations in the ACA. The findings reveal that the pooled mean length of the A1 segment is 14.47 mm, while the pooled mean diameter is 2.00 mm. For the A2 segment, the pooled mean lengths were 18.39 mm for the right ACA and 18.34 mm for the left ACA, with a pooled mean diameter of 1.76 mm. Notably, the analysis also identified significant anatomical variations, with an overall pooled prevalence of 1.32% for the AzACA, 2.65% for the MedACA, and 1.12% for the BihemACA. It is hoped that the results of the present study will assist clinicians and neurosurgeons in managing various cerebrovascular disorders and procedures, ultimately enhancing the safety and outcomes of these interventions.

## Data Availability

All the data is included in the article.

## Abbreviations

The following abbreviations are used in this manuscript:

ACA	Anterior cerebral artery
AzACA	Azygos ACA
BihemACA	Bihemispheric ACA
MedACA	Median ACA
CTA	Computed Tomography Angiography
DSA	Digital Subtraction Angiography
MRA	Magnetic Resonance Angiography
MRI	Magnetic Resonance Imaging
